# Evidence-based leakage management in cancer-preventive ileostomy care: A Delphi consensus integrating systematic review and clinical expertise

**DOI:** 10.1016/j.apjon.2025.100703

**Published:** 2025-04-21

**Authors:** Songxian Zhao, Xueling Ma, Yujue Wang, Yan Bai, Chongyu Yan

**Affiliations:** a Ostomy Nursing Clinics, Tianjin Medical University Cancer Institute & Hospital, National Clinical Research Center for Cancer, Tianjin's Clinical Research Center for Cancer, Tianjin Key Laboratory of Digestive Cancer, Tianjin, China; b Department of Colorectal Cancer, Tianjin Medical University Cancer Institute & Hospital, National Clinical Research Center for Cancer, Tianjin's Clinical Research Center for Cancer, Tianjin Key Laboratory of Digestive Cancer, Tianjin, China

**Keywords:** Ileostomy care, Peristomal skin complications, Evidence-based practice, Delphi consensus, Leakage prevention, Ostomy nursing

## Abstract

**Objective:**

This study aimed to synthesize the best available evidence and integrate clinical expertise to develop a structured, evidence-based leakage management system for cancer-preventive ileostomy care.

**Methods:**

A two-phase mixed-methods design was employed. First, a systematic review was conducted following the PIPOST framework (Population: cancer-preventive ileostomy patients; Intervention: leakage management strategies; Professionals: ostomy caregivers; Outcomes: leakage incidence and skin complications; Setting: hospitals; Evidence types: clinical decisions, guidelines, evidence summaries, best practices, systematic reviews, and expert consensus). Databases and clinical repositories were searched from inception to June 2024, yielding 23 high-quality documents. Second, a Delphi consensus process involving 15 ostomy nursing experts across six Chinese provinces refined the evidence into actionable clinical protocols through two iterative consultation rounds. Consensus thresholds included a Likert score ≥ 4 and coefficient of variation < 0.25.

**Results:**

The finalized leakage management system comprises three domains—prevention, assessment, and intervention—organized into 11 themes and 46 actionable items. Key components include preventive strategies for ileostomy leakage, dynamic ostomy appliance selection, and protocols for managing leakage-related skin damage. Expert consensus highlighted the importance of individualized care, with adjustments based on effluent characteristics and gas production. The Delphi panel achieved high agreement (*Cr* ​= ​0.89, *Kendall's W* ​= ​0.194–0.137, *P* ​< ​0.05).

**Conclusions:**

This study presents a robust, evidence-based leakage management system tailored to the needs of cancer-preventive ileostomy survivors. By integrating high-quality evidence with practical clinical insights, the system offers valuable guidance for improving patient outcomes and enhancing the quality of ostomy nursing care in real-world settings.

**Systematic review registration:**

ES20245104.

## Introduction

Rectal cancer is one of the most common malignant tumors, and anastomotic leakage is a serious and potentially life-threatening complication that has a 6% to 26% mortality rate^1^^,^^2^ after rectal cancer surgery. Preventive ileostomy is conventionally performed to prevent anastomotic leakage in clinical practice. Related large-sample studies have shown that the implementation rate of ileostomy is between 52.8% and 89%.^3^, ^4^, ^5^

Preventive ileostomy has unique excretion characteristics. The output is watery, highly alkaline, and contains proteolytic enzymes and the initial daily defecation volume of the new ostomy is approximately 1200 mL, even after a certain period of intestinal adaptation, the daily defecation volume remains at approximately 500–800 mL.^6^ In addition, study has shown that the incidence of high-output ileostomy, which involves a daily defecation volume of approximately 1500–2000 mL can reach 23%.^7^ Consequently, prolonged exposure of peristomal skin to ileostomy effluent increases chemical irritation, leading to maceration and compromised epidermal integrity, which may result in ulceration.^8^^,^^9^

Ostomy leakage is commonly used to describe ostomy effluent that reaches beyond the baseplate or spreads underneath the baseplate.^10^ It is not only a major problem itself but also the cause of many problems. Leakage or fear of leakage has a severe impact on daily and social activities, as do emotional effects that appear to last up to one year after the last leakage incident.^11^^,^^12^ Compared with those with colostomy, ileostomy patients are more likely to have ostomy management-related difficulties, especially leakage and leakage-related peristomal skin complications, and the uncomfortable feeling of patients is more obvious.^13^ Our previous qualitative study^14^ also confirmed that patients with ileostomy face complex symptoms (especially those related to high-output ileostomy), generally avoid social interactions (particularly due to concerns about public leakage), and experience significant disruptions to their sleep patterns.

In recent years, medical professionals and scholars have increasingly focused on ileostomy leakage. Existing evidence indicates^15^ that ileostomy leakage is associated with a marked reduction in patients’ quality of life. However, there remains a lack of systematic management frameworks in this area, research has often been confined to single-dimensional perspectives. Previous studies 16, 17, 18 identified multiple factors influencing leakage in cancer ileostomy patients, including age, gender, body mass index (BMI), and diabetes mellitus. Furthermore, patients undergoing radiotherapy or chemotherapy are more likely to develop a high-output ileostomy,^7^ which increases the risk of leakage due to prolonged chemical irritation of peristomal skin. When assessing and managing leakage, nurses often rely on traditional nursing practices. However, new assessment tools and interventions have emerged, such as peristomal body profile assessment,^19^ patient-reported tool^12^ and even artificial intelligence technology assessment.^20^ The efficacy of different ostomy systems in reducing leakage risk varies.^21^ A randomized controlled trial (RCT) even utilized emerging 3D printing technology to customize ostomy systems to prolong the wearing time and reduce leakage incidence.^22^ The proliferation of novel research findings has undoubtedly challenged conventional nursing approaches.

In the context of clinical practice, specialized ostomy nurses not only need to continuously follow the dynamic changes and updates of knowledge, but also have to take into account the diverse and complex individualized needs of patients. This undoubtedly poses numerous challenges to the management of ileostomy leakage. When faced with complex clinical situations, unidimensional knowledge frameworks are insufficient to address the complexities of decision-making and provide effective leakage management strategies, a systematic management system is urgently needed. Therefore, this study synthesizes the best available evidence and incorporates expert opinions to develop an ileostomy leakage management system to improve patient quality of life and provide reference for clinical practice.

## Methods

### Evidence summary

#### Problem formulation

The research question was formulated via the PIPOST model^23^: “P” (population): cancer preventive-ileostomy patients; “I” (intervention): leakage management strategies; “P” (professionals): ostomy caregivers; “O” (outcomes): leakage incidence and skin complications; “S” (setting): hospitals; “T” (types of evidence): clinical decisions, guidelines, evidence summaries, best practices, systematic reviews, and expert consensus.

The evidence summary was registered on the Fudan University Centre for Evidence-based Nursing Platform in Shanghai, China. The registration number is ES20245104.

#### Search strategy

Following the “6S” classification model for evidence-based resources, a comprehensive search was conducted on 15 clinical decision websites and guideline websites, 15 professional association websites and 10 databases. For each website and database, a pre-search was conducted to confirm the applicable subject words and free words. The search time limit was from the establishment of the database to June 30, 2024. Details of search strategies are presented in Table 1.

**Table 1:** Details of search strategies.

Search sources		Search strategies

Clinical decision and guideline websites	1. BMJ best practice	**Subject words and free words:** ostomy or enterostomy or “surgical stoma” or ileostomy or “fecal ostomy” or “intestinal ostomy” or “temporary stoma” or “diverting ostomy” or “defunctioning stoma” or “defunctioning ostomy” or stoma **Searching steps at PubMed:** #1 “Ostomy”[Mesh]) OR “Enterostomy”[Mesh]) OR “Ileostomy”[Mesh]) OR “surgical Stomas”[Mesh] #2 “fecal ostomy” [Ti/Ab] OR “intestinal ostomy” [Ti/Ab] OR enterostom∗[Ti/Ab] OR “temporary stoma” [Ti/Ab] OR “temporary enterostom∗” [Ti/Ab] OR “temporary ileostom∗”[Ti/Ab] OR “protective enterostom∗” [Ti/Ab] OR “protective ileostom∗” [Ti/Ab] OR “diverting ostomy” [Ti/Ab] OR “defunctioning stoma” [Ti/Ab] OR “defunctioning ostomy” [Ti/Ab] OR (stoma [Ti/Ab] #3 #1 OR #2 #4 “practice guideline” [publication type]) OR “Consensus”[Mesh]) OR “evidence-based Nursing”[Mesh]) OR “systematic review” [publication Type] #5 “clinical practice guideline”[Ti/Ab] OR “expert consensus”[Ti/Ab] OR “evidence summary”[Ti/Ab] OR “evidence based practice”[Ti/Ab] OR “best practice”[Ti/Ab] OR “recommended practice”[Ti/Ab] #6 #4 OR #5 #7 #3 AND #6
	2. Up to date	
	3. YiMaiTong clinical guide network	
	4. Chinese medical ace base	
	5. Practice guideline registration for transparency (PREPARE)	
	6. Guideline international network, GIN	
	7. Registered nurses' association of Ontario, RNAO	
	8. National institute for health and care excellence, NICE	
	9. National comprehensive cancer network, NCCN	
	10. The agency for health care research and Quality's, AHRQ	
	11. Scottish intercollegiate guidelines network, SIGN	
	12. Canadian medical Association's clinical practice guidelines database, CPG InfoBase	
	13. Cancer Australia	
	14. National health and medical research council of Australia, NHMRC	
	15. New Zealand guidelines group, NZGG	
Associations	1. The world council of enterostomal therapists, WCET	
	2. Wound, ostomy and continence nurses society, WOCN	
	3. United ostomy associations of America, UOAA	
	4. HONG KONG ENTEROSTOMAL THERAPISTS ASSOCIATION	
	5. American society of colon and rectal surgeons, ASCRS	
	6. Nurses specializing in wound, ostomy and continence Canada, NSWOCC	
	7. American cancer society, ACS	
	8. American gastroenterology association, AGA	
	9. American society of clinical oncology, ASCO	
	10. Oncology nursing society, ONS	
	11. European society for medical oncology, ESMO	
	12. American nurses association, ANA	
	13. CHINESE NURSING ASSOCIATION	
	14. CHINA ANTI-CANCER ASSOCIATION	
	15. World health organization	
Databases	1. CNKI	
	2. WANFANG DATA	
	3. WEIPU	
	4. SinoMed	
	5. PubMed	
	6. Cochrane Library	
	7. JBI EBP database	
	8. CINAHL	
	9. EMBASE	
	10. Web of science	

JBI, Joanna Briggs Institute; BMJ, British Medical Journal.

#### Evidence inclusion and exclusion criteria

The inclusion criteria were as follows: the research population was adult patients with cancer who had undergone ileostomy; the research content involved the content explicitly related to enterostomy leakage; the research types included publicly published guidelines, evidence summaries, clinical decisions, best practices, expert consensuses, and systematic reviews; and full-text articles in Chinese or English.

The exclusion criteria were as follows: the literature type was guideline interpretation, duplicate publication, updated/translation version of existing works; studies with incomplete information or unavailable full-text materials; and research involving pediatric enterostomy patients or non-cancer populations.

#### Literature quality assessment criteria

The guidelines were evaluated via the Appraisal of Guidelines for Research and Evaluation II (AGREE II).^24^ Systematic reviews and expert consensuses were evaluated using different literature types from the Joanna Briggs Institute (JBI).^25^ Quality evaluations of the clinical decisions and evidence summaries were traced back to the original studies. The JBI evaluation criteria were selected based on the original research type. Among them, Studies from authoritative institutions such as Up to Date, BMJ (British Medical Journal) Best Practice, and JBI were directly included. Two researchers independently evaluated the data. In cases of disagreement, a group discussion was conducted to reach a consensus.

#### Evidence synthesis

Two authors extracted and integrated evidence from the included literature and independently assigned evidence grades. After discussion with the evidence-based team, the evidence system was preliminarily determined. We conducted the next stage to make the leakage management system more consistent with the clinical situation.

### Delphi survey

#### Expert selection

We selected 15 experts specializing in ostomy nursing management, ostomy clinical nursing, and ostomy nursing clinics from six Chinese provinces (Tianjin, Hebei, Hubei, Guizhou, Hainan, and Heilongjiang) for the consultation.

The eligibility criteria were as follows: experts with at least 10 years of professional experience in the field, professional titles at or above the intermediate level, and voluntary participation.

#### Delphi procedure

The first Delphi consultation contained two parts: expert status and the preliminarily formed system. Participants were first asked to provide demographic information, followed by rating the importance of each evidence-based item using a five-point Likert scale (1 ​= ​unimportant, 5 ​= ​very important). Additionally, experts were required to explain their reasoning if they thought that specific content should be deleted or added. A space was left after each scenario and the experts were requested to include additional written comments to justify or qualify their scores.

The second Delphi round was conducted to achieve a consensus on the content. The experts were provided with the results of the previous round and instructed to consider these results when completing the new questionnaire of the present round. The experts were provided with the following information: (a) the mean scores of other experts for each item, (b) any additional comments made by other experts, and (c) the contents of the modifications.

The questionnaires were distributed and recovered via e-mail and in person during the two rounds of Delphi consultations. Each expert received a personal invitation with the study protocol, including research-based information and an explanation of why they should not discuss the study with colleagues to ensure anonymity. The experts did not know who was invited to participate. Each round continued until no further answers were obtained.

### Statistical analysis

We used the questionnaire recovery rate to reflect expert positivity. The experts' familiarity with problem (*Cs*) and basis for judgment (*Ca*) determine their authoritative coefficient (*Cr*), that is, Cr=(Ca+Cs)÷2. The *Coefficient of Variation* and *Kendall's coefficient of concordance* (*Kendall's W*) reflect the degree of coordination among the experts' opinions. The cutoff point for deletion was an importance rating below 4 (5-point Likert scale). A consensus level was determined by selecting an expert *Coefficient of Variation* of less than 0.25.

## Results

### Evidence summary

**Fig. 1:**
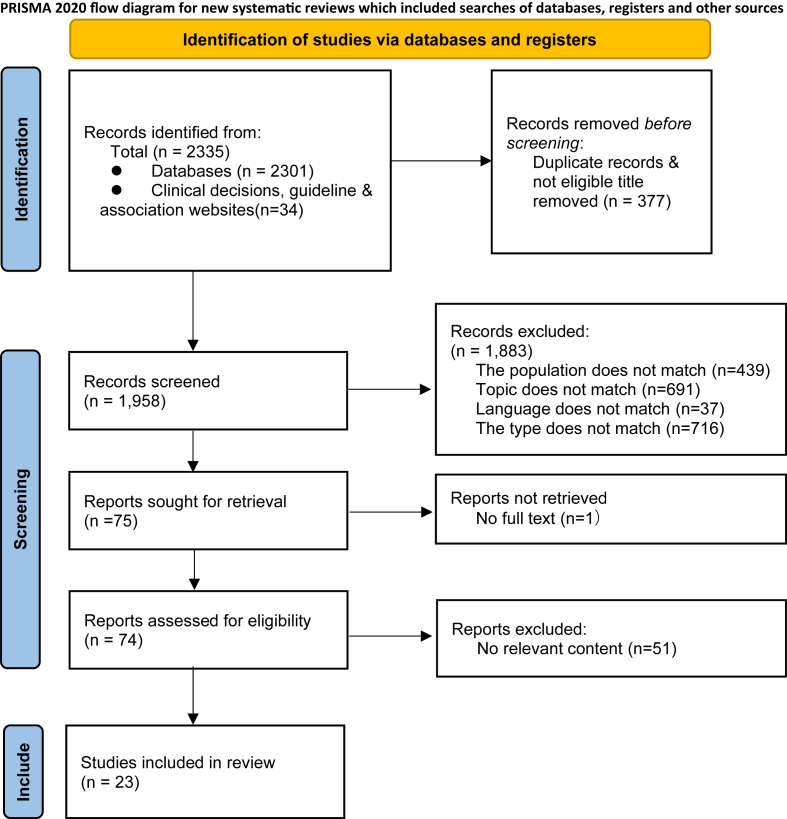
Flow diagram illustrating the original process of screening and identification of studies*.*

A total of 2335 studies were obtained through comprehensive retrieval. After deduplication using Endnote, screening by reading titles and abstracts, and screening by reading the full text, 23 articles were included. The screening process is illustrated in Fig. 1. The general characteristics of the included studies are presented in Table 2. The quality assessment of guidelines and consensus are presented in Table 3 and Table 4 respectively. Only one systematic review that met the inclusion criteria was included, and all items were evaluated as “yes”. Studies from Up to Date and JBI were directly included. Through evidence extraction and integration, we preliminarily formed a leakage management system for cancer-preventive ileostomy patients.

**Table 2:** General characteristics included in the study (*N* ​= ​23).

Author	Literature source	Publication date	Literature type	Literature title	Main related themes

Francone TD, et al.^26^	UpToDate	2023	Clinical decision	Overview of surgical ostomy for fecal diversion	Ileostomy localization
Landmann RG, et al.^27^	UpToDate	2024	Clinical decision	Ileostomy or colostomy care and complications	Perioperative education Leakage prevention strategies Decision-making on ostomy supplies High output ileostomy
WOCN^28^	WOCN	2015	Guideline	Peristomal skin complications: Clinical resource guide	Leakage assessment Treatment of skin damage
ASCN^29^	ASCN	2016	Guideline	Stoma care National clinical guidelines	Leakage assessment Treatment of leakage and skin damage
WOCN^30^	WOCN	2018	Guideline	Management of the adult patient with a fecal or urinary ostomy-an executive summary	Perioperative education Decision-making on ostomy supplies, leakage assessment and management
RNAO^31^	RNAO, registered nurses' association of Ontario	2019	Guideline	Supporting adults who anticipate or live with an ostomy	Perioperative education Ileostomy localization
WCET^32^	WCET	2020	Guideline	International ostomy guideline	Perioperative education Ileostomy localization Leakage assessment and management
Perrin A, et al.^33^	CINAHL	2021	Guideline	Convexity in stoma care: developing a new ASCN UK guideline on the appropriate use of convex products	Decision-making on ostomy supplies Leakage assessment Treatment of leakage due to different reasons
Davis BR, et al.^34^	The american society of colon and rectal surgeons	2022	Guideline	Clinical practice guidelines for ostomy surgery	Perioperative management
Aubert M, et al.^35^	YiMaiTong	2024	Guideline	Management of adult intestinal stomas	Decision-making on ostomy supplies specific management of ileostomy
Colwell JC, et al.^36^	PubMed	2011	Expert consensus	Peristomal moisture-associated dermatitis and periwound moisture-associated dermatitis	Decision-making on ostomy supplies Leakage assessmen Treatment of leakage and skin damage
Gray M, et al.^37^	CINAHL	2013	Expert consensus	Peristomal moisture-associated skin damage in adults with fecal ostomies	Leakage assessment Specific management of ileostomy
Colwell JC, et al.^38^	CINAHL	2019	Expert consensus	Development of practice guidelines for assessment of peristomal body and stoma profiles, patient engagement, and patient follow-up	Decision-making on ostomy supplies Leakage assessment
Ratliff CR, et al.^39^	Wound, ostomy, and continence nurses society	2021	Expert consensus	Peristomal skin health: A WOCN society consensus conference	Ileostomy leakage prevention strategies Decision-making on ostomy supplies Specific management of ileostomy Leakage assessment
Ostomy professional committee, Chinese society of coloproctology et al.^40^	Chinese medical ace base	2022	Expert consensus	Chinese expert consensus on protective ostomy for mid⁃low rectal cancer (version 2022)	Ileostomy localization Specific management of ileostomy
Colwell JC, et al.^41^	CINAHL	2022	Expert consensus	Use of a convex pouching system in the postoperative period	Decision-making on ostomy supplies
Hedrick TL, et al.^42^	American gastroenterological association	2023	Expert consensus	AGA clinical practice update on management of ostomies: Commentary	Causes and management of ostomy leakage
The Chinese Ostomy Collaboration Group et al.^43^	Chinese medical ace base	2023	Expert consensus	Criteria of enterostomy complications: Classification and grading (2023 edition)	High output ileostomy
Ambe PC, et al.^44^	PubMed	2022	Systematic review	The effect of preoperative stoma site marking on risk of stoma-related complications in patients with intestinal ostomy	Ileostomy localization
Yimei li, et al.^45^	JBI	2018	Evidence summary	Stoma: Care and assessment	Perioperative education
DONG Shan, et al.^46^	CNKI	2022	Evidence summary	Summary of the best evidence for the prevention and management of peristomal moisture-associated skin damage in adults	Ileostomy leakage prevention strategies Leakage assessment Specific management of ileostomy Treatment of skin damage
Overall, et al.^47^	JBI	2022	Evidence summary	Stoma care: Preoperative site markings	Ileostomy localization
Magtoto, et al.^48^	JBI	2023	Evidence summary	Stoma care: Post-operative patient assessment and education	Perioperative education Ileostomy leakage prevention strategies

WOCN, wound, ostomy and continence nurses society; JBI, Joanna Briggs Institute.

**Table 3:** Quality evaluation results of included guidelines (*N* ​= ​8).

Study	Standardized scores in various domains (%)						Quality
	Scope and purpose	Stakeholder involvement	Rigor of development	Clarity of presentation	Applicability	Editorial independence	
WOCN^28^	100.0	72.2	65.6	86.1	54.2	54.2	B
ASCN^29^	100.0	80.6	78.1	83.3	70.8	79.2	A
WOCN^30^	94.4	86.1	78.1	88.9	56.3	79.2	B
RNAO^31^	100.0	97.2	91.7	97.2	85.4	91.7	A
WCET^32^	100.0	94.4	90.6	100.0	85.4	91.7	A
Perrin A, et al.^33^	97.2	75.0	52.1	61.1	66.7	66.7	B
Davis BR, et al.^34^	88.9	83.3	82.3	80.6	56.3	79.2	B
Aubert M, et al.^35^	97.2	91.7	85.4	86.1	70.8	75.0	A

WOCN, wound, ostomy and continence nurses society; ASCN, Association of Stoma Care Nurses; RNAO, Registered nurses' association of Ontario; WCET, world council of enterostomal therapists.

**Table 4:** Quality evaluation results of included expert consensus (*N* ​= ​8).

Evaluation	Item 1∗	Item 2∗	Item 3∗	Item 4∗	Item 5∗	Item 6∗	Overall appraisal
Colwell JC, et al.^36^	Yes	Yes	Yes	Unclear	Yes	No	Included
Gray M, et al.^37^	Yes	Yes	No	Yes	Yes	No	Included
Colwell JC, et al.^38^	Yes	Yes	Yes	Unclear	Yes	Unclear	Included
Ratliff CR et al.^39^	Yes	Yes	Yes	Yes	Yes	No	Included
Ostomy professional committee, Chinese society of coloproctology et al.^40^	Yes	Yes	Yes	Yes	Yes	No	Included
Colwell JC, et al.^41^	Yes	Yes	No	Yes	Yes	No	Included
Hedrick TL et al.^42^	Yes	Yes	No	Yes	Yes	No	Included
The Chinese Ostomy Collaboration Group et al.^43^	Yes	Yes	No	Yes	Yes	No	Included

Item 1∗: Is the source of the opinion clearly identified? Item 2∗: Does the source of opinion have standing in the field of expertise? Item 3∗: Are the interests of the relevant population the central focus of the opinion? Item 4∗: Does the opinion demonstrate a logically defended argument to support the conclusions drawn? Item 5∗: Is there reference to the extant literature? Item 6∗: Is any incongruence with the literature/sources logically defended?.

### Delphi survey

#### Experts’ general information and the reliability of expert advice

Influential experts in this field comprised the expert group (*n* ​= ​15). The age of the experts was (44.9 ​± ​6.3) years. The length of working years is (23.6 ​± ​8.2) years. Details are presented in Table 5. All the invited experts agreed to participate in the study. The active coefficients of the experts in both rounds of Delphi consultation were 100%. The *Ca* and *Cs* were 0.95 and 0.82, respectively, and the average *Cr* was 0.89. The *Kendall's W* coefficients in the two rounds of Delphi consultation were 0.194 and 0.137, respectively, and the *P* values were less than 0.05. The experts' opinions were consistent.

**Table 5:** Characteristics of experts (*N* ​= ​15).

Variables	*n* (%)
Gender	
Male	0 (0.00)
Female	15 (100.00)
Age (years)	
< 30	0 (0.00)
31–40	3 (20.00)
41–50	9 (60.00)
> 50	3 (20.00)
Working experience (years)	
10–20	7 (46.67)
21–30	4 (26.67)
> 30	4 (26.67)
Educational background	
Bachelor's degree	13 (86.67)
Master's degree	2 (13.33)
Title	
Intermediate level	7 (46.67)
Associate senior level	6 (40.00)
Senior level	2 (13.33)
Field of specialization	
Ostomy nursing clinics	7 (46.67)
Ostomy clinical nursing	4 (26.67)
Ostomy nursing management	4 (26.67)
Enterostomal therapists	
Yes	15 (100.00)
No	0 (0.00)

#### Consultation opinions from the experts

Data from the experts were collected in two rounds over four months. All experts (100%) responded to the questionnaire. Five experts (33.3%) provided opinions, and they all reached a consensus in the second round. The modified details were as follows: (1) Combined with expert opinions and group discussions, we believe that the frequency of ostomy system replacement should not be based solely on how many days a one-piece ostomy system can be worn or how many days a two-piece ostomy system can be worn. It is important to dynamically adjust the frequency of ostomy system replacement according to the shape of defecation and the peristomal skin. (2) Experts noted that gas production is an important factor leading to ostomy leakage. After group discussion, we added “gas production” to the cause of leakage. (3) In theory, medical treatment measures should be incorporated in the management of high-output ileostomies. However, considering the care scope of enterostomal therapists in China and the application object of this research, we briefly described drug treatment related to high-output ileostomy and did not elaborate. The final leakage management system contains three first-level themes, 11 second-level themes, and 46 specific contents. The details are shown in Table 6.

**Table 6:** The leakage management system for cancer preventive ileostomy.

Themes		Mean score	SD score	Variation
I	Leakage prevention	5.00	0.00	0.00
I-1	Perioperative education	4.80	0.41	0.09
I-1-1	Perioperative education is targeted at patients and their caregivers and is ensured by a team of professional medical staff. Health service organizations should provide access to nurses specialized in wound, ostomy, and continence as essential members of the interprofessional team for all persons who anticipate or live with an ostomy.^31^^,^^34^	4.87	0.35	0.07
I-1-2	Provide supportive counseling for patients before surgery, conduct in-depth counseling for patients who are difficult to adapt, and educate patients as much as possible before surgery to prepare for life with a temporary ostomy. Preoperative education includes instructions on surgical methods, the impact of surgery on quality of life, expectations for ileostomy reversal, ostomy, and ostomy care.^27^^,^^30^^,^^32^	4.60	0.63	0.14
I-1-3	Postoperative education should last from the first day after the operation until the patient has the corresponding ability, and the self-care ability of the ostomy should be evaluated before discharge.^45^^,^^48^	4.93	0.26	0.05
I-2	Ileostomy localization	4.87	0.35	0.07
I-2-1	The location of the ostomy is related to the significant reduction of leakage risk,^44^ and should be first selected by an enterostomal therapist, wound, ostomy and continence nurse, or ostomy care specialist, and also could be performed by a surgeon.^26^^,^^27^^,^^31^^,^^47^	4.80	0.41	0.09
I-2-2	The following principles should be followed when positioning the ostomy^26^^,^^40^: (1) routinely perform preoperative positioning; (2) consider the patient's peristomal body profile, previous abdominal incisions, bony protrusions, occupation, clothing requirements, disabilities, and any physical limitations, and examine the patient from the standing, sitting, and from the front and side (especially obese individuals); (3) the line connection method or the triangle positioning method can be chosen, and the ileostomy should be located in the rectus abdominis, which should be at least 5 cm away from all skin folds, scars, old incisions, waistlines, umbilicus, and bony processes. (4) special patients, such as those with a deformed spine column, scars on the lower abdomen, or severe radiation treatment injury, should choose an ostomy position according to the situation of abdominal; patients with short tunica and small intestines that are difficult to drag out from the right lower abdomen can choose the left lower abdomen for ostomy positioning; for patients with obesity and BMI ≥ 30 kg/m^2^, the ostomy position should be set at the highest position of abdominal bulge; (5) it is recommended to locate multiple, at least two. The position should be protected by a marker pen that is not easy to fade or protected by a transparent film, and is kept completely visible in the operating room.	4.87	0.35	0.07
I-3	Ileostomy leakage prevention strategies	4.93	0.26	0.05
I-3-1	The best strategy for leakage management is prevention. Enterostomal therapists or ostomy nurses implement leakage prevention and management plans, and encourage patients and their caregivers to participate in ostomy care together.^32^^,^^42^^,^^48^	5.00	0.00	0.00
I-3-2	The replacement frequency of the ileostomy system can refer to one-piece replacement every 1–3 days, two-piece replacement 2–3 times a week, and the wearing time is not more than 7 days. When there is a sign of leakage or the peristomal skin feels itchy and burning, it can be replaced at any time.^29^^,^^46^	4.80	0.41	0.09
I-3-3	Ostomy size should be measured at each appliance change for the first 8 weeks. Except for special cases such as ostomy prolapse or ostomy edema, the opening of the ostomy chassis should be closely attached to the root of the ostomy (the junction of skin and mucosa).^39^^,^^42^	4.87	0.35	0.07
I-3-4	When removing the chassis, use both hands to slowly remove it from top to bottom at a low angle, and at the same time flatten the skin at the edge of the viscous contact surface between skin and chassis to avoid skin damage.^39^	4.73	0.46	0.10
I-3-5	Use scissors to cut off the hair around the ostomy or a disposable razor to shave the hair around the ostomy in the direction of hair growth. Avoid using depilatory or dry shaving and consider permanent hair removal technology.^39^	4.53	0.52	0.11
I-3-6	The best way to clean the skin around the ostomy is to clean it regularly with ordinary soft materials such as paper, towels and water, and avoid the use of alkaline soap and unnecessary use of sterile products.^39^	4.60	0.63	0.14
I-3-7	Ileostomy should routinely use barrier rings or paste to ensure that the skin is not exposed to drainage. Spray and wipe adhesive remover are recommended.^27^^,^^46^	4.73	0.46	0.10
I-4	Decision-making on ostomy supplies	4.93	0.26	0.05
I-4-1	The appropriate and effective ostomy system should be selected by wound, ostomy and continence nurses or enterostomal therapists together with patients.^30^^,^^41^	4.80	0.41	0.09
I-4-2	The selection of ostomy products should not be based on the provider's preference, fixed order (such as starting with the flat product, and then changing to the convex product if the result is not satisfactory), or reverse repeat testing to determine. The following factors should be considered: The surgery type, ostomy type and position, abdominal profile, education level, lifestyle, cognitive status, visual acuity, hand dexterity, skin assessment results, and social factors related to patients, such as housing, economic conditions, access to health care/supplies, members of vulnerable groups, and racial influences. Tools can also be used to select ostomy supplies.^30^^,^^38^^,^^39^	4.73	0.46	0.10
I-4-3	A tailorable and plastic chassis should be used within 6 weeks after surgery until the ostomy size is stable. If the ostomy is irregular, it is recommended to continue to use. If the product is replaced, the sealing condition, patient wearing time, and acceptance of the new system should be re-evaluated within 2–3 weeks after replacement. If the patient has weight fluctuations, re-evaluate the effectiveness of the ostomy system.^36^^,^^39^^,^^41^	4.60	0.51	0.11
I-4-4	The using conditions of convex ostomy supplies^27^^,^^30^^,^^34^^,^^41^: The wearing time of the ostomy system is not ideal, wrinkles or creases appear in the surrounding area of the ostomy when sitting or standing, ostomy retraction, ostomy opening is flush with or lower than the surrounding skin, an ostomy is located in the abdominal retraction, skin around the ostomy is relaxed, patients have repeated leakage, skin complications around the ostomy due to leakage, there are ravines or scars around the ostomy, the abdomen is soft, and the surrounding area of the ostomy is pulled inward or concave into the abdomen.	4.73	0.46	0.10
I-4-5	The following considerations should be taken into account when using convex products: (1) detailed and comprehensive individualized evaluation by professional ostomy nurses before use^33^; (2) convex chassis requires a relatively flat, at least 4 ​cm of skin paste range, and away from scars, skin folds, and bone protrusions^27^; (3) the convex products could be considered after surgery, but the type and characteristics of the convex products used should be based on the ability to provide safe, stable and predictable sealing and exert minimum pressure on the junction of the skin and mucosa. It is generally recommended to use the minimum convex depth and place it near the ostomy to ensure effective pressure. If the convex chassis alone cannot provide a safe seal, the ostomy belt should be added, but it should be noted that the use of the belt immediately after the operation may increase the pressure on the skin–mucosa junction^33^^,^^35^^,^^41^; (4) make sure that patients understand the reasons for using the convex ostomy and can use it correctly. The possible negative effects of using the convex ostomy should also be pointed out. If any skin changes (such as bruises and indentations) are found, professional ostomy nurses should be contacted.^33^	4.67	0.62	0.13
I-4-6	Customized convex products are also an option for difficult cases.^36^	4.40	0.63	0.14
II	Leakage assessment	5.00	0.00	0.00
II-1	Leakage assessor	4.80	0.41	0.09
II-1-1	Professional ostomy nurses are most suitable for assessing and solving problems and complications related to ostomy care.^33^ After the leakage occurs, the ostomy nurse should select a private and safe environment and obtain the oral consent of the patient, then evaluate the cause of the leakage and record it with photos if possible.^29^	4.80	0.41	0.09
II-2	Leakage assessment timing	4.73	0.46	0.10
II-2-1	The adhesive surface of the chassis should be checked every time the ostomy system is removed or replaced. It is considered to be of clinical significance when the adhesive erosion range under the chassis exceeds 1/8 inch.^30^^,^^37^	4.73	0.46	0.10
II-3	Leakage assessment tool	4.67	0.49	0.10
II-3-1	Based on understanding the characteristics of healthy skin, standardized description and monitoring of the skin around the ostomy are performed through visual inspection and evaluation tools.^37^^,^^38^ Visual examination first focuses on the color and integrity of the skin, followed by the location, shape, size, and distribution of skin irritation or skin immersion range.^36^ The evaluation tools included^29^^,^^46^ studio alterazoni cutanee stomale, the ostomy skin tool, and the DET tool. The tool should also be used to communicate with patients and their caregivers.^32^	4.60	0.51	0.11
II-4	Leakage assessment contents	4.80	0.41	0.09
II-4-1	Leakage causes^29^: poor adhesion of ostomy chassis, stoma and skin flush or retraction, skin folds or creases, consistency and amount of excreta, ostomy gas production, and other factors such as skin diseases, ulcers, bleeding, wounds, etc.	4.80	0.41	0.09
II-4-2	Patient characteristics^28^^,^^33^^,^^36^^,^^37^: (1) general factors: Including postoperative time, medical history and medical status, age, medication, diet and fluid management, cognitive and physical ability, vision and hand flexibility, obesity, weight change and patient perception of the problem; (2) Behavioral factors: Including heating the chassis with a microwave oven, not wearing an ostomy bag for some time, exposing the ostomy to air, reinforcing rather than replacing the leaking chassis, the method of using/removing the ostomy system by the patient or caregiver, skin cleaning and care techniques, and wearing the ostomy chassis for more than the recommended 3–7 days; (3) Lifestyle factors: Living alone or avoiding any assistance in ostomy care, contact with external water sources (hot baths, baths, swimming), sports and recreational activities.	4.67	0.49	0.10
II-4-3	Abdominal profile^28^^,^^33^^,^^38^: (1) when the patient is lying, sitting, bending, and standing, observe the abdominal area to determine whether the skin has superficial wrinkles and deep wrinkles; (2) determine the location of the ostomy relative to the following areas: Body shape, bony protrusions, umbilicus, skin folds, scars, any signs of hernia, abdominal distension, other medical equipment ; (3) abdominal muscle tension, firm or relaxed; (4) the texture (soft, hard) and shape (regular, inward or outward) of the abdominal area around the ostomy.	4.87	0.35	0.07
II-4-4	Ileostomy^28^^,^^38^: Evaluate the shape, location (above, at, or below the waistline), height of the excretory orifice (above, at, or below the skin level around the ostomy), type and characteristics of excreta, and high-output ostomy-related symptoms.	4.87	0.35	0.07
II-4-5	High output ileostomy^27^^,^^29^^,^^43^: Excretory volume > 1500 mL/d, lasting ≥ 3 d, the discharge was water-like, the frequency of ostomy bag emptying increased, patients had thirst, lethargy, weakness, muscle weakness, dry mucosa, decreased skin elasticity, weight loss, and other symptoms and signs.	4.80	0.56	0.12
II-4-6	Ostomy system^36^^,^^37^: One-piece or two-piece, chassis size and type, whether to obtain and use the required products, the use of accessories, damage/leakage, ostomy bag replacement frequency and emptying method, chassis wearing time and removal method.	4.73	0.46	0.10
II-4-7	Leakage performance^43^: The chassis mucous bubble expands, and the skin under the chassis has an itching or burning sensation. Long-term stimulation can cause skin inflammatory lesions and damage, mainly manifested as skin redness, swelling, pain, and increased skin temperature.	4.67	0.49	0.10
II-4-8	The skin around the ostomy^39^: The healthy skin around the ostomy is characterized by integrity, color, and texture similar to the adjacent and contralateral abdominal skin, without inflammation, itching, burning, or pain.	4.67	0.62	0.13
III	Leakage intervention	5.00	0.00	0.00
III-1	Specific management of ileostomy	4.80	0.41	0.09
III-1-1	Develop and implement skin protection programs to reduce the source of moisture, including^28^^,^^46^: (1) reducing the source of moisture; (2) isolating damp source and skin; (3) protecting the skin.	4.80	0.56	0.12
III-1-2	The methods to promote effective sealing include^36^^,^^37^^,^^46^: (1) it is recommended to use a durable chassis for ileostomy, which cannot be strengthened after leakage occurs; (2) limit or avoid the behavior that may interfere with the sealing between the chassis and the skin around the ostomy; (3) guide the patient to develop a chassis replacement schedule and continuously monitor its sealing.	4.80	0.41	0.09
III-1-3	Transparent, closed, and drained ostomy bags were used immediately after ileostomy to observe the daily ostomy discharge. For patients with high excretory volume, high-volume ostomy bags should be used for continuous drainage, and the skin around the ostomy should be checked for signs of damage while monitoring their urine volume and weight.^29^^,^^39^^,^^35^^,^^40^	4.67	0.62	0.13
III-1-4	The high output ileostomy needs to be managed by formulating and implementing nursing pathways, programs, or plans. The measures that can be taken include^29^^,^^30^^,^^35^: Finding the cause, ensuring that patients wear appropriate ostomy supplies, using fiber supplements according to doctor's advice, and drug treatment.	4.67	0.49	0.10
III-1-5	In the case of long-term loose stools or high output, ileostomy requires additional fluid intake or regulation of fecal viscosity,^39^ limiting oral hypotonic or hypertonic fluids (tea, coffee, juice, or soda water) for rehydration, and reducing food intake by patients that will increase their ostomy output.^35^	4.60	0.51	0.11
III-1-6	Considered for early reversal of the ostomy and restoration of intestinal continuity, when feasible.^27^	4.40	0.74	0.17
III-2	Treatment of leakage due to different reasons	4.87	0.52	0.11
III-2-1	Treatment measures for poor adhesion of the ostomy chassis include^36^: Re-measuring the ostomy to ensure accurate cutting of the chassis opening, thoroughly cleaning the skin and shaving the hair, wiping the remaining adhesive, using leakage prevention products, observing and guiding patients to cut and use the chassis, and using other supplies.	4.67	0.62	0.13
III-2-2	The treatment measures for leakage caused by ostomy flat, retraction, skin wrinkles or creases include: Using appropriate convex products after evaluation by ostomy nurses (soft convex is preferred for solid abdomen around ostomy, hard convex is preferred for soft abdomen), ostomy belt, barrier rings or paste to fill grooves or make ostomy excretory ostomy prominent.^29^^,^^33^^,^^42^	4.87	0.35	0.07
III-2-3	The treatment measures for leakage caused by changes in stool traits include: Determining the cause and treating it, and adding a curing agent in the ostomy bag.^29^	4.33	0.82	0.19
III-2-4	The treatment measures for leakage caused by skin condition changes, skin diseases, ulcers, and bleeding include: Assessing skin condition and treatment options, using antiperspirants to control sweating, seeking medical advice, using silver nitrate or cryotherapy to treat granulomas, and checking drug use (such as immunosuppressants, steroids).^29^	4.60	0.63	0.14
III-2-5	Refractory cases of ostomy leakage may require ostomy revision.^42^	4.53	0.74	0.16
III-3	Treatment of skin damage caused by leakage	5.00	0.00	0.00
III-3-1	Actively treat any minor skin damage to prevent deterioration.^27^	4.80	0.41	0.09
III-3-2	After skin injury, it needs to be treated according to the evaluation results. The treatment initially focused on changing the type of ostomy system and the frequency of replacement.^36^	4.67	0.49	0.10
III-3-3	A small amount of ostomy powder was gently applied to the injured area, and a non-irritating liquid skin protective film was sprayed until dry to provide protection.^46^	4.73	0.46	0.10
III-3-4	Skin damage is large or exudate is more, hydrocolloid dressing can be considered.^28^	4.47	0.83	0.19
III-3-5	The skin condition should be recorded using the ostomy skin tools.^29^	4.67	0.49	0.10
III-3-6	In severe cases, doctors should be consulted and steroid sprays should be used to reduce inflammation. If there is a fungal infection, nystatin powder can be used.^28^	4.60	0.51	0.11

SD, standard deviation; BMI, body mass index.

## Discussion

### Roles of prevention strategies, key assessment points and active interventions in managing ileostomy leakage among cancer patients

The best strategy for leakage management is prevention,^42^ a series of predictive measures should be taken to avoid the occurrence of leakage, specifically including: perioperative education, ileostomy localization, ileostomy leakage prevention strategies and decision-making on ostomy supplies. The Registered Nurses’ Association of Ontario^31^ and the American Society of Colon and Rectal Surgeons^34^ emphasize that perioperative education should be ensured by a team of professional medical staff, among which wound, ostomy, and continence nurses play an important role as team members. Implementing ileostomy localization is related to the significant reduction of leakage risk.^44^ Selecting an effective barrier pouch is one of the top concerns of people living with an ostomy,^38^ and notably, barrier rings or paste should routinely be used to ensure that the skin is not exposed to drainage among ileostomy patients.^27^ However, currently, the number of specialized wound, ostomy, and continence nurses is limited, and in addition to wound and ostomy care, they also undertake a large amount of clinical nursing work,^49^ which restricts the comprehensive implementation of measures such as ostomy positioning and decision-making on ostomy supplies. Therefore, in clinical practice, it is necessary to strengthen team building, improve the role division and collaboration model, and implement targeted preventive measures according to the individual conditions of patients to enhance nursing care quality.

In this study, leakage assessment refers to the evaluation of leakage manifestations and leakage-related factors, including: leakage assessor, timing, tool and specific contents. The Association of Stoma Care Nurses UK points out^33^ that professional ostomy nurses are best suited to assess and address ostomy-related complications. Experts emphasize that key assessment components include the evaluation of the abdominal profile^28^^,^^33^^,^^38^ and the ileostomy characteristics,^28^^,^^38^ as inappropriate ostomy products directly contribute to leakage, the assessment of the abdominal profile directly affects the selection of the most suitable product type for the patient. In clinical practice, it is recommended to observe the abdominal area in multiple positions (lying, sitting, bending, standing) to identify superficial/deep wrinkles, locate the ostomy relative to bony prominences or skin folds, and evaluate muscle tension and the texture (soft/hard) and shape (regular/inward/outward) of the peristomal skin. Current research often groups colostomy and ileostomy together, overlooking the unique characteristics of ileostomy—factors that increase the risk of leakage and subsequent skin damage. Medical staff should therefore prioritize evaluating ileostomy-specific attributes (shape, location, height of the excretory orifice, effluent type and characteristics, and high-output symptoms) and adopt patient-centered approaches to conduct comprehensive and effective leakage assessments.

Active management of ileostomy leakage and associated minor skin damage can effectively prevent deterioration.^27^ In this study, leakage intervention includes: specific management of ileostomy, treatment of leakage due to different reasons and treatment of skin damage caused by leakage. Many guidelines^29^^,^^30^^,^^35^ advocate for the development and implementation of standardized nursing pathways, plans, or programs for ileostomy care. For cases of high ileostomy output, it is critical to identify the leakage etiology, ensure proper ostomy appliance selection, prescribe fiber supplements, and administer medications as clinically indicated. However, the absence of systematic management frameworks and standardized protocols^50^ contributes to recurrent leakage and varying degrees of skin damage. In face of such situations, nursing staff often use ostomy powder and hydrocolloid dressings for treatment. Notably, high ileostomy output and repeated leakage are associated with risks of complications beyond local damage, such as electrolyte imbalance^29^ and renal dysfunction,^43^ which require close monitoring by health care providers. In addition to paying attention to the leakage symptoms, it is also necessary to deeply explore the root causes of leakage, so as to prevent severe complications. To improve care quality, there is a need to establish standardized management protocols that promote continuous quality improvement in leakage management.

### Implications for nursing practice and research

This study provides a structured, evidence-based system for ileostomy leakage management, enabling nurses to standardize proactive prevention, precise evaluation, and tailored interventions in clinical practice. The 46 specific items support developing practice guidelines, nursing training curricula, and clinical pathways. For future research, randomized controlled trials could assess its long-term impact on patient quality of life, expand the study population, and address intra-group variations and inter-group care differences to inform holistic strategies for patients.

### Limitations

This study has several limitations that require acknowledgment. First, the evidence summary relies on existing literature, which may be affected by publication bias and heterogeneous research quality. Second, individual characteristics such as age, physical activity levels, and comorbidities are inadequately considered, limiting the generalizability of findings across complex scenarios. Additionally, pediatric ostomy patients are excluded, as their unique physiological, psychological, and treatment response traits necessitate specialized techniques, supplies, and child-centered care.

## Conclusions

In this study, we developed a scientific and effective leakage management system for cancer-preventive ileostomy to meet clinical needs. For ileostomy patients and health care providers, the system plays a very important role in reducing the leakage burden, helping patients adapt to the life of ileostomy, and optimizing evidence-based clinical care practices.

## CRediT authorship contribution statement

**Songxian Zhao:** Conceptualization, Methodology, Formal analysis, Writing – Original Draft. **Yujue Wang**: Data Curation, Methodology, Formal analysis. **Yan Bai**: Conceptualization, Data curation. **Chongyu Yan**: Conceptualization, Data curation. **Xueling Ma**: Conceptualization, Supervision, Writing – Review & Editing. All authors have read and approved the final manuscript.

## Ethics statement

Not required.

## Funding

This study was supported by Tianjin Key Medical Discipline (Specialty) Construction Project (Grant No. TJYXZDXK-011A). The funders had no role in considering the study design or in the collection, analysis, interpretation of data, writing of the report, or decision to submit the article for publication.

## Data availability statement

The data that support the findings of this study are available from the corresponding author, XM, upon reasonable request.

## Declaration of generative AI and AI-assisted technologies in the writing process

No AI tools/services were used during the preparation of this work.

## Declaration of competing interest

The authors declare no conflict of interest.
